# YY1 is regulated by ALKBH5-mediated m6A modification and promotes autophagy and cancer progression through targeting ATG4B

**DOI:** 10.18632/aging.205037

**Published:** 2023-09-18

**Authors:** Shijiang Wang, Jiangbo Nie, Kaiying Xu, Yangyang Liu, Weilai Tong, Anan Li, Wei Zuo, Zhili Liu, Feng Yang

**Affiliations:** 1 Department of Orthopedic Surgery, The First Affiliated Hospital of Nanchang University, Nanchang 330006, People’s Republic of China; 2 Medical Innovation Center, The First Affiliated Hospital of Nanchang University, Nanchang 330006, People’s Republic of China; 3 Institute of Spine and Spinal Cord, The First Affiliated Hospital of Nanchang University, Nanchang 330006, People’s Republic of China; 4 Department of Thoracic Surgery, The First Affiliated Hospital of Nanchang University, Nanchang 330006, People’s Republic of China; 5 Department of Oncology, The First Affiliated Hospital of Nanchang University, Nanchang 330006, People’s Republic of China; 6 Postdoctoral Innovation Practice Base, The First Affiliated Hospital of Nanchang University, Nanchang 330006, People’s Republic of China

**Keywords:** YY1, autophagy, cancer, ATG4B, N6-methyladenosine

## Abstract

YY1 affects tumorigenesis and metastasis in multiple ways. However, the function of YY1 and the potential mechanisms through which it operates in gastric cancer (GC) progression by regulating autophagy remains poorly understood. This study aimed to assess the essential transcription factors (TFs) involved in autophagy regulation in GC. Western blot, RFP-GFP-LC3 double fluorescence and transmission electron microscopy (TEM) assays were used to probe autophagy activity in GC cells. Methylated RNA immunoprecipitation (MeRIP) was utilized to evaluate the ALKBH5-regulated m6A levels of YY1. Gain- and loss-of-function assays were employed in the scrutiny of the biological effects of the ALKBH5/YY1/ATG4B axis on cancer cell proliferation and invasion abilities *in vitro*. Per the findings, YY1 was identified as a crucial transcriptional activator of cancer autophagy-related genes and promoted the proliferation and aggressiveness of cancer cells associated with enhanced ATG4B-mediated autophagy. However, ectopic ALKBH5 expression abolished the YY1-induced effect via m6A modification. Importantly, YTHDF1 facilitated the mRNA stability of YY1 through m6A recognition. Collectively, this study found that YY1 was regulated by ALKBH5 and YTHDF1-mediated m6A modification and served as an autophagy-dependent tumor driver to accelerate cancer progression through ATG4B transactivation, providing an exploitable therapeutic target for GC.

## INTRODUCTION

Autophagy is a process of intracellular degradation by lysosomal hydrolases to digest integrated proteins, damaged organelles or pathogens, producing fatty acids, sugars, nucleotides, amino acids and ATP [1–3]. Basal autophagy provides capacity and ingredients by eliminating damaged cellular components to maintain cellular metabolism and survival during starvation [4]. Dysregulating autophagy is critical to cancer progression. Emerging evidence supports the dynamism of autophagy-mediated effects in cancer, depending on the type, stage or genetic environment [5]. For instance, autophagy can maintain genomic stability and suppress the accumulation of the p62 protein, hence preventing proliferation and metastasis at the early stages of cancer development [6]. Autophagy can also act as a cytoprotective agent by boosting mitochondrial functioning and reducing DNA damage, ultimately facilitating cancer cell resistance to therapeutic agents [7].

During autophagosome formation, cytoplasmic microtubule-associated protein 1 LC3B-I is converted to LC3B-II in a phosphatidylethanolamine-coupled form [8], making LC3B a widely used marker for monitoring autophagy. As a bridge molecule between polyubiquitin proteins and LC3B, P62 can selectively be encapsulated in autophagosomes and then degraded by autolysosomes [9]. Increased LC3B and decreased P62 expressions are associated with advanced autophagy activity and poor clinical features in many cancer types [10, 11]. Therefore, exploring the specific role and mechanisms of autophagy is of significant importance for effective autophagy-based cancer treatment. However, the key regulator of the program of autophagic machinery genes in gastric cancer (GC) is yet to be identified.

Yin-Yang 1 (YY1), a ubiquitously expressed and multifunctional zinc-finger transcription factor [12], plays a crucial role in many biological processes, including cancer cell progression. YY1 can either promote or inhibit target gene expression, depending on its interaction partners, and it is also the primary driver behind the epigenetic network in cancer [13]. For example, YY1 is enriched in the promoters of CCDC43 and ADRM1 genes and facilitates GC proliferation and metastasis [14]. In addition, YY1 positively regulates TNK2-AS1 and exacerbates osteosarcoma development by “sponging” miR-4319 and elevating WDR1 [15]. Furthermore, YY1 binds with PLIC11 to promote lung cancer proliferation and metastasis via the stimulation of PIWIL4 transcription and expression [16]. Previous studies have shown that increased YY1 expression is associated with worse clinical features in many cancers [17–19]. Notably, in a recent study, YY1 was proven to enhance autophagy by suppressing miR-30a expression in pancreatic cancer cells [20]. Although autophagy is hugely significant in tumor development, the underlying mechanisms of how YY1 operates in the regulation of autophagy in GC remain undetermined.

M6A (N6-methyladenosine) modification, the most prevalent internal reversible modification process in eukaryotic mRNAs [21], is mediated by the “writer” of METTL3, METTL14, and WTAP, eliminated by the “eraser” of FTO or ALKBH5, and preferentially identified by m6A “reader” [22]. The m6A “reader” proteins bind to m6A-modified mRNAs in the cytoplasm, and regulate cellular processes and physiological functions [23]. M6A reportedly participates in almost every RNA metabolism step, including mRNA splicing, export, folding, degradation and translation [24, 25]. Remarkably, RNA m6A-related genes act as pivotal regulators that promote the development and progression of various tumors, including lung cancer, liver cancer, osteosarcoma, and breast cancer [26]. Recently, ALKBH5 was found to decrease mRNA m6A levels in human osteosarcoma cells, thereby inhibiting cell proliferation [27]. Additionally, m6A modifications ease YY1 mRNA stability. For example, METTL3-mediated m6A modifications strengthen YY1 mRNA stability, resulting in the accelerated growth of multiple myeloma cells [28]. However, the underlying mechanisms through which m6A regulators are involved in YY1-induced autophagy have not been fully elucidated yet.

This investigation confirmed YY1 as a crucial regulator that facilitates autophagy and GC progression through ATG4B transactivation. Notably, ALKBH5 knockdown reduced YY1 demethylation, while YTHDF1 boosted its m6A methylation recognition sensitivity and mRNA stabilization, eventually heightening YY1 expression. In terms of mechanism, YY1 was regulated by ALKBH5 and YTHDF1-mediated m6A modifications and activated the ATG4B-dependent autophagic pathway, leading to the promotion of GC progression. Overall, the YY1-induced autophagic pathway was established as a potential target for the treatment of GC.

## MATERIALS AND METHODS

### Cell culture

Human gastric cancer cells (MGC803, AGS, MKN45 and BGC823) and normal human gastric mucosal cells (GES-1) were purchased from the American Type Culture Collection and Nanjing Cobioer Biosciences (China). All cells were cultured with 10% fetal bovine serum (FBS), 100 u/ml streptomycin and 100 μg/ml penicillin under standard conditions (5% CO 2 at 37°C).

### Real-time quantitative PCR (RT-qPCR)

First, total RNA was isolated using the EZ-press RNA Purification Kit (B0004D), and was then reverse transcribed to cDNA with a common product (Vazyme, R212-01, China). Real-time quantitative PCR was performed using the SYBR qPCR Master Mix kit. Primers used for qPCR were synthesized by Biotech (Shanghai, China) as described in Supplementary Table 1. ACTB mRNA was used as control. The relative expression of the target gene was determined utilizing the 2^−ΔΔct^ method.

### Western blot

Proteins were extracted from cells using the RIPA lysis buffer (Thermo Fisher Scientific, USA) and then separated with SDS-PAGE before being transferred to PVDF membranes (Millipore, USA). Next, the proteins on the PVDF membranes were incubated with primary antibodies for LC3B (CST, #3868), SQSTM1/p62 (CST, #23214), Beclin-1 (CST, #3738), YY1 (CST, #46395), ATG4B (CST, #13507), ALKBH5 (CST, #80283), and YTHDF1 (CST, #57530) before visualization using the Bio-Rad chemiluminescence system.

### CRISPR/dCas9-mediated inhibition and activation

To activate or inhibit gene expression, the CRISPR/dCas9 approach was employed [29, 30]. Independent gRNAs targeting YY1, ALKBH5, YTHDF1 and ATG4B (Supplementary Table 2) were inserted into engineered CRISPRi and CRISPRa plasmids. Briefly, the wild type Cas9 coding sequence (Cas9 CDS) in the lentiCRISPR v2 plasmid was mutated to yield the dead Cas9 (dCas9) protein with D10A and H840A mutations and, hence, no endonuclease activity. Next, the BFP-KRAB domain obtained from pHR-SFFV-dCas9-BFP-KRAB (Addgene, #46911) and the VPR domain copied from SP-dCas9-VPR (Addgene, #63798) were subcloned to the C-terminus of dCas9 to produce the CRISPRi (Ci) and CRISPRa (Ca) constructions. Briefly, in this dCas9 system, infectious lenti-viruses supernatants were harvested from HEK-293T cells about 60 hours later after co-transfection of the cells with CRISPRa/i, psPAX2 and pMD2G plasmids at the same time. The virus supernatants were filtered with the PVDF filter (0.22 μm Millipore, USA), centrifuged at 12000 g for 10 minutes, and then stocked in a final volume of 100–200 μl. For the CRISPRi or CRISPRa screen, cancer cells were infected with the single or pooled lenti-viruses. 72 hours later, cancer cells were treated with puromycin (Invitrogen, USA), and after 2–3 weeks of puromycin selection, cells were collected and western blot was applied to verify efficiency of gene overexpression or knockdown, thus stable cell lines were obtained.

### Data mining and analysis

The autophagy-related genes (222 of them) were downloaded from the Human Autophagy Database (Supplementary Table 3). The R package “Rcis Target” and the BRAT website (http://bartweb.org/) were used to identify the key transcription factors that regulate autophagy (Supplementary Table 3). The RNA sequencing data and clinical survival data of 375 gastric tumor patients and 32 precancerous tissues were downloaded from Xena (http://xena.ucsc.edu). The correlations between YY1 and other genes were determined using the Pearson correlation analysis.

### Cell viability and proliferation assays

The CCK-8 solution was added directly to each well of all the tumor cells (2 × 10^3^ per well) seeded in 96-well plates, and the mixture was incubated for 2 h. OD values were measured at 450 nm using Enzyme Markers. For the colony formation assay, 2 × 10^3^ infected cells were maintained in each well, and colonies were fixed with 4% paraformaldehyde and then stained with 0.1% crystalline violet. Colonies containing more than 50 cells were counted.

### Migration assays

Cell migratory capacity was gauged employing the transwell assay. Briefly, 2 × 10^4^ cells were inoculated into the upper chamber after resuspension with serum-free medium, and 20% FBS medium was added to the lower chamber before incubation was initiated. After 24 h of incubation, the cells were fixed and then stained with 0.1% crystalline violet (Sigma, USA). Migrated cells were counted and imaged.

### MeRIP (Methylated RNA immunoprecipitation)

Cells were mixed with the RIP lysis buffer and immunoprecipitated by co-incubation with an m6A antibody using a Magna methylated RNA immunoprecipitation m6A Kit (Millipore, USA). The enrichment of mRNA containing m6A was then analyzed utilizing RT-qPCR.

### Immunohistochemistry

Paraffin-embedded tumor tissues were cut into 4-mm sections, flattened in water at 45°C, and baked at 70°C for 30 min. Antibodies specific for Ki-67 (Cell Signaling Technology, #9449; 1:200 dilution) were then introduced, and incubation was initiated. In the end, the intensity was imaged and measured.

### Confocal microscopy

Tumor cells were inoculated on glass-bottom culture dishes, infected with RFP-GFP-LC3 lentivirus for 48 h, fixed in 4% paraformaldehyde, and then stained with DAPI for nuclei scrutiny. Ultimately, the autophagic flux was monitored using confocal laser scanning microscopy (STELLARIS 5, Leica, USA).

### Dual-luciferase reporter assay

The promoter of ATG4B was subcloned into a pGL3-basic vector, and the 3′-UTR of YY1 mRNA was inserted into the psiCHECK2 plasmid. Dual-luciferase assay was performed following instructions (Promega, USA), and the promoter activity of ATG4B was normalized to the Firefly/Renilla ratio, while the 3′-UTR activity of YY1 was normalized to the Renilla/Firefly ratio. Primers used here are listed in Supplementary Table 2.

### ChIP assay

The ChIP assay was conducted using the ChIP assay kit (Beyotime Institute Biotechnology, P2078, China) per the manufacturer’s protocol. YY1-immunoprecipitated DNA was PCR amplified using specific primers, and the YY1 binding site of the putative ATG4B promoter was analyzed. Primers used here are listed in Supplementary Table 1.

### RNA stability assays

Cells were incubated with actinomycin D (HY-17,559, MCE) for 0 h, 2 h, 4 h or 6 h before RNA collection. qRT-PCR was employed using the primers listed in Supplementary Table 1 to detect the half-life of YY1 mRNA.

### Xenografts in mice

Five-week-old male BALB/c nude mice were used for tumor growth studies *in vivo*. Briefly, AGS cells were subcutaneously injected into the dorsal side of mice blindly and randomly (*n* = 5 per group). Tumor volume was measured twice a week and calculated as follows: V = W^2^ × L/2. All animal experiments were performed in accordance with the NIH Guidelines for the Care and Use of Laboratory Animals and approved by the ethics committee of the First Affiliated Hospital of Nanchang University.

### Statistical analysis

GraphPad Prism 8.0.2 (USA) was used for data analysis, with the resulting data presented as the mean ± standard error of the mean (SEM). Statistical tests were two sided. A value of *P* < 0.05 was considered statistically significant.

### Availability of data and materials

The data supporting the conclusions of this article are included in this article and its additional files.

## RESULTS

### YY1 is up regulated and promotes autophagy in GC

**Figure 1:**
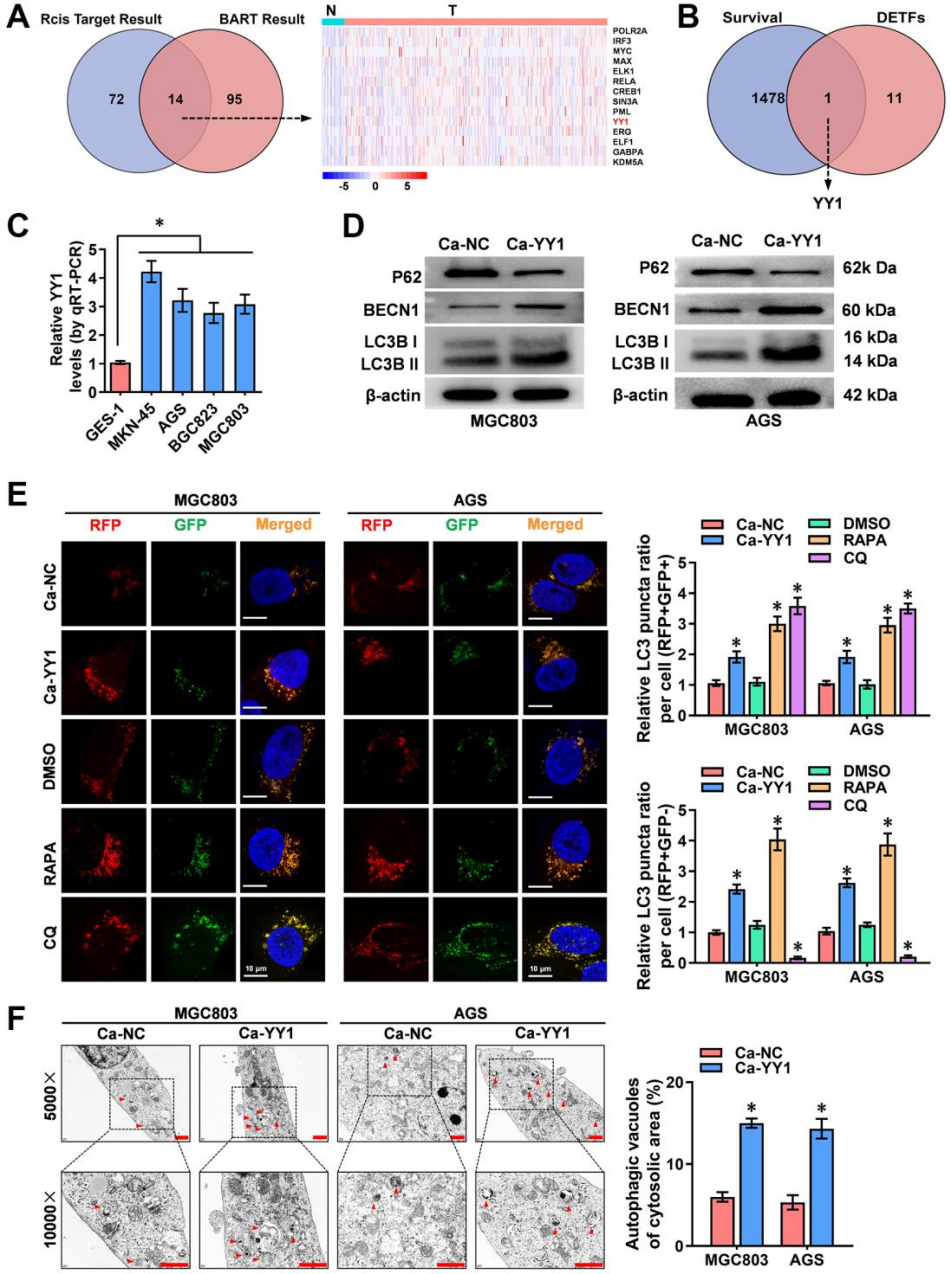
**YY1 is upregulated and promotes autophagy in cancer.** (**A**) Venn diagram indicating overlapped genes from two publicly available bioinformatics analyses. The right panel shows gene expression features in cancer and adjacent tissues from the Xena platform. (**B**) Overlapping analysis (Venn diagram) revealing the 12 TFs with survival-related genes. (**C**) qRT-PCR analysis showing the expression of YY1 in GC cell lines. (**D**) Western blotting displaying the expression of autophagy-associated proteins in AGS and MGC803 cells treated with the empty vector (Ca-NC) or Ca-YY1. (**E**, **F**) Immunofluorescence staining and TEM scanning demonstrating fluorescence intensity and autophagic vacuoles in AGS and MGC803 cells treated with the empty vector (Ca-NC) or Ca-YY1, and those treated with DMSO, CQ (Chloroquine, 20 μmol/L) or RAPA (Rapamycin, 1 μmol/L) as positive controls for inactivated or activated autophagy. Scale bar: 10 μm in E and 1 μm in F. ^*^P < 0.05 vs. GES-1, Ca-NC, DMSO.

To identify the key transcription factors (TFs) that regulate autophagy, the 222 autophagy-related genes downloaded from the Human Autophagy Database (HADb, http://www.autophagy.lu) were subjected to upstream TF analysis using the R package “Rcis Target” and BRAT website (http://bartweb.org), and 14 TFs were identified (Figure 1A and Supplementary Table 1). Next, the expression levels of the obtained 14 autophagic genes-regulating TFs were evaluated in the acquired tumor and normal sample RNA sequencing data and clinical survival data of 375 GC patients and 32 precancerous tissues, respectively, downloaded from Xena (http://xena.ucsc.edu) (Figure 1A and Supplementary Figure 1). The Cancer Genome Atlas (TCGA) public database was used for analyzing the survival values of gene expression in GC patients. Through overlapping analysis, YY1 was identified as the only TF associated with both poor prognosis and enhanced autophagy activity in GC (Figure 1B).

To assess the expression of YY1 in GC cells, RT-qPCR was performed, with the results revealing significantly higher presence of YY1 in the cancer cells than in the normal GES-1 cells (Figure 1C). To further validate the regulatory role of YY1 on autophagy, the impact of YY1 overexpression or knockdown was scrutinized using the dCas9-based Clustered regularly interspaced short palindromic repeats (CRISPR) interference or activation (CRISPRi, Ci; CRISPRa, Ca) methods [11, 29]. Two independent gRNAs against YY1 (Ca-YY1, Ci-YY1) designed from the ZHANG LAB website (https://zlab.bio/guide-design-resources) and introduced into GC cell lines were used to examine the efficiency, and the superior Ca-YY1 #2- and Ci-YY1 #1-mediated performances of promotion or inhibition were selected for the subsequent gain-and-loss evaluation (Supplementary Figure 2A, 2B). To observe the regulatory effects of YY1 on autophagy in GC cells, western blot, RFP-GFP-LC3 double fluorescence with confocal microscopy and transmission electron microscopy (TEM) were applied. As shown in the results, there was an increase in the autophagy-specific markers of LC3B and BECN1 and a decrease in the expression of P62 in MGC-803, AGS, MKN-45 and BGC823 cells with YY1 overexpression (Figure 1D and Supplementary Figure 2C). According to the autophagy flux assay, YY1 promoted both the autophagosome (yellow fluorescence, RFP+GFP+) and autolysosome (red fluorescence, RFP+GFP-) formation in MGC-803, AGS, MKN-45 and BGC823 cells (Figure 1E and Supplementary Figure 2D). TEM analysis validated the elevated number of autophagic vacuoles in MGC-803 and AGS cells with stable YY1 overexpression transfection (Figure 1F). These results suggested that YY1 facilitated autophagy in GC.

### YY1 promotes cancer progression through autophagy enhancement

**Figure 2:**
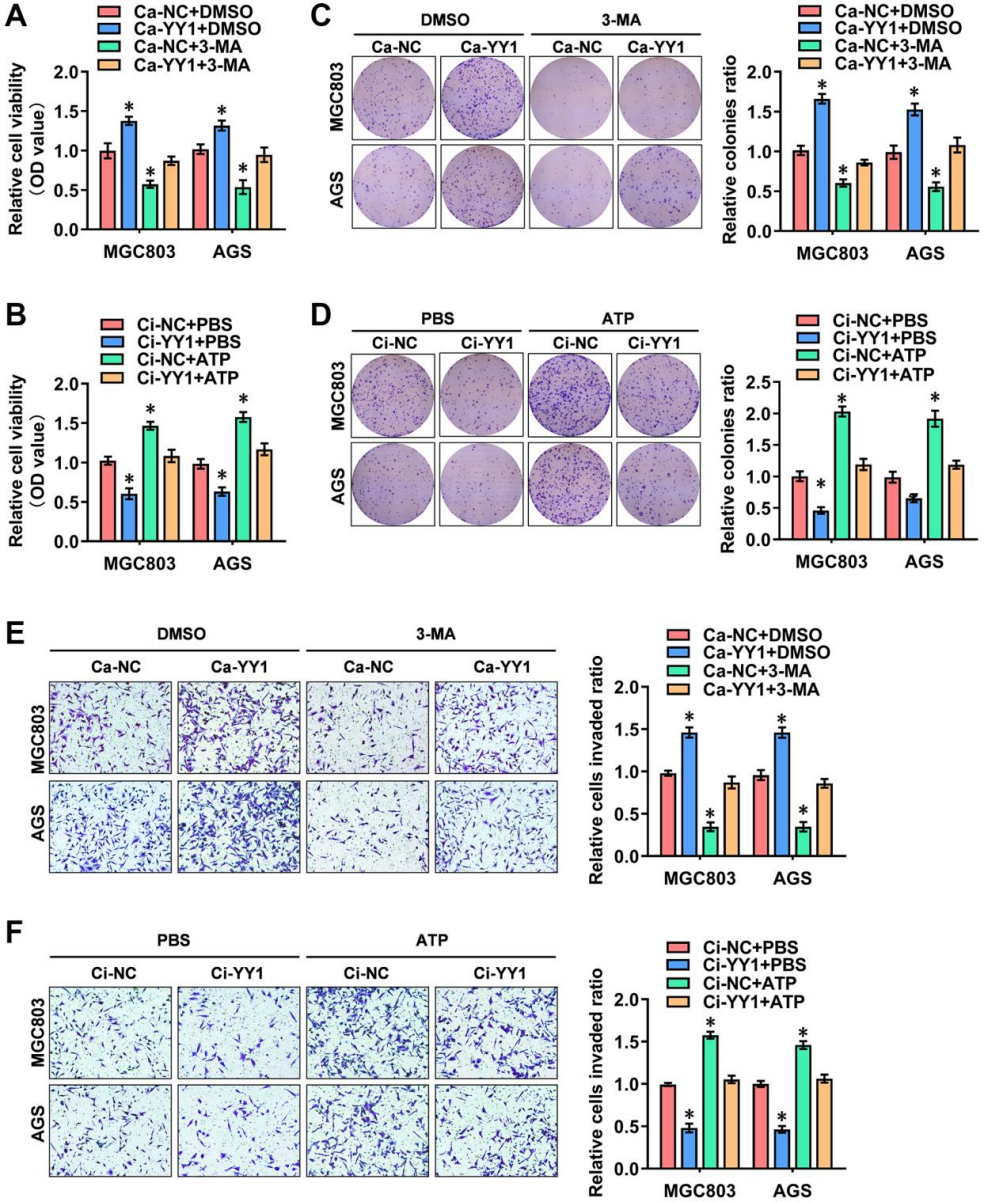
**YY1 promotes cancer progression through autophagy enhancement.** (**A**, **B**) The *in vitro* CCK-8 assay of AGS and MGC803 cells stably transfected with Ca-NC, Ca-YY1, Ci-NC or Ci-YY1 and those treated with DMSO, 3-MA (1.0 μM), PBS or ATP (0.1 mM). (**C**–**F**) Representative images (left) and the quantification (right) of colony formation (**C**, **D**) and transwell (**E**, **F**) assays showing the growth and migration of GC cells transfected with Ca-NC, Ca-YY1, Ci-NC or Ci-YY1 and those treated with 3-MA (1.0 μM) or ATP (0.1 mM). ^*^P < 0.05 vs. Ca-NC+DMSO, Ci-NC+PBS.

The possible association between the oncogenic function of YY1 and autophagy was investigated. For this purpose, the autophagy inhibitor of 3-methyladenine (3-MA) and the agonists of adenosine triphosphate (ATP) were added to detect the cellular function of YY1 in GC. The CCK-8 (Figure 2A, 2B), colony formation (Figure 2C, 2D) and transwell assays (Figure 2E, 2F) all showed that YY1 presence promoted the viability, proliferation and migration activity of cancer cells, while the inhibition of YY1 resulted in the opposite outcome. Notably, 3-MA or ATP incubation-mediated autophagy inhibition or activation abolished these cellular effects, respectively. These findings suggested that YY1 promoted GC progression in an autophagy-dependent manner *in vitro*.

### YY1 facilitates ATG4B expression

**Figure 3:**
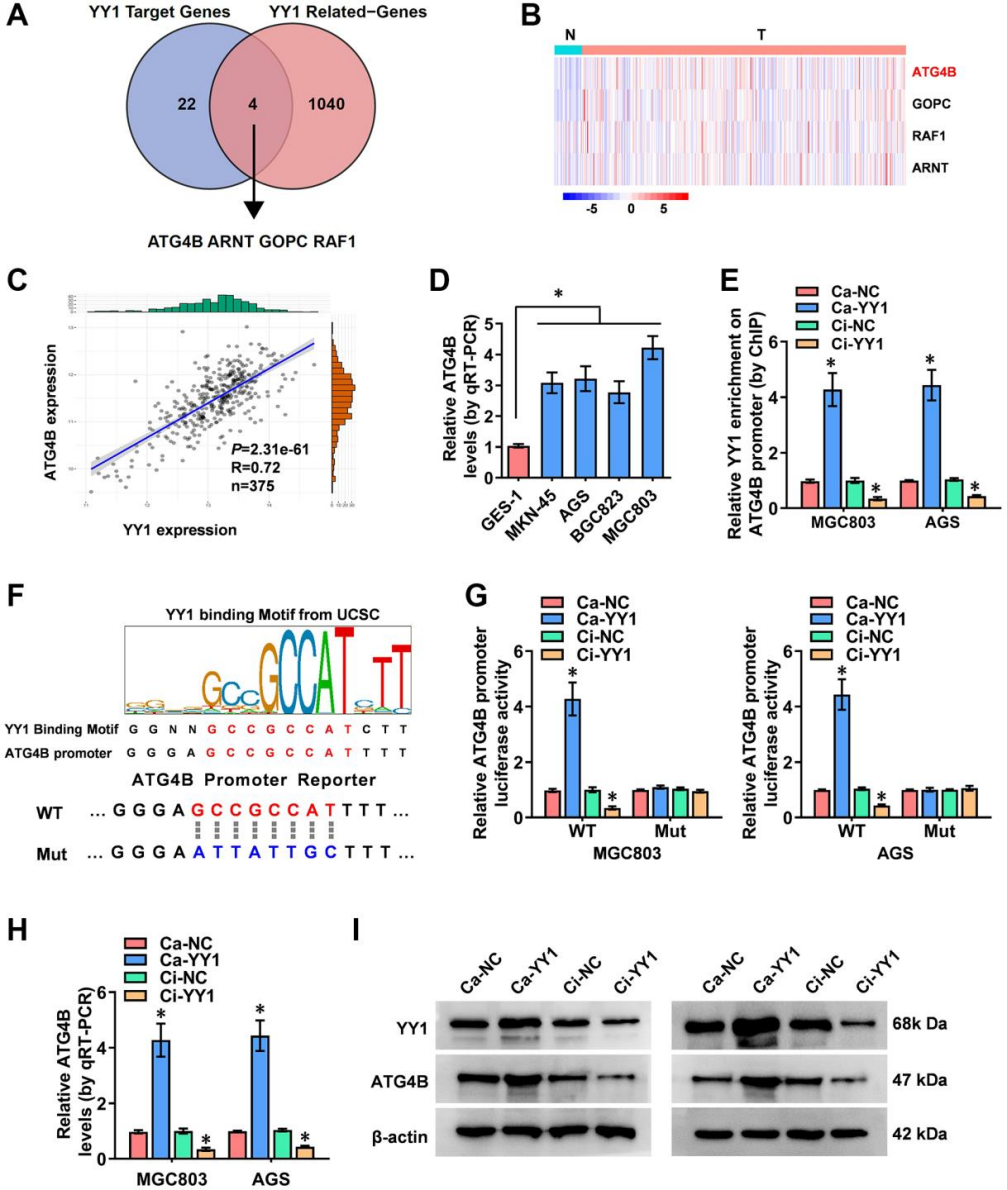
**YY1 facilitates ATG4B expression.** (**A**) The analysis of the overlap (Venn diagram) between YY1 target genes and YY1-related genes revealing the four targets involved in the YY1 regulation of autophagy-related genes. (**B**) Heatmap showing the expression features of those four genes in GC and adjacent normal tissues from the Xena platform. (**C**) A correlation test validating the association between ATG4B and YY1 on the Xena platform. (**D**) qRT-PCR assay displaying the expression levels of ATG4B in GC and GES-1 cells. (**E**) ChIP assay unveiling YY1 enrichment on the ATG4B promoter in MGC803 and AGS cells stably transfected with Ca-NC, Ca-YY1, Ci-NC or Ci-YY1. (**F**) Schematic illustration showing the YY1 binding sites on the ATG4B promoter at -135/-121 bases from UCSC Genome Browser, and the wild-type (WT) or mutations (Mut) of ATG4B promoter reporters were designed. (**G**) Dual-luciferase assay disclosing the luciferase activity of ATG4B promoter in AGS and MGC803 cells stably transfected with Ca-NC, Ca-YY1, Ci-NC or Ci-YY1. (**H**, **I**) qRT-PCR and western blot assays showing the transcript and protein expression levels of ATG4B in MGC803 and AGS with YY1 overexpression or knockdown. ^*^P < 0.05 vs. GES-1, Ca-NC, Ci-NC.

Comprehensive analyses to elucidate the underlying mechanisms of YY1-induced autophagy in GC were conducted next. Through the overlapping analysis of 26 YY1 target genes from the Human Autophagy Database and 1044 YY1-related genes from the UCSC Xena platform (data source: TCGA database), ATG4B (autophagy-related 4B cysteine peptidase), ARNT (aryl hydrocarbon receptor nuclear translocator), GOPC (golgi-associated PDZ and coiled-coil motif containing) and RAF1 (Raf-1 proto-oncogene) were identified as the potential targets of YY1-mediated autophagy activation (Figure 3A and Supplementary Table 4). The expression features of those four genes in GC and the adjacent normal tissues were, therefore, evaluated, and ATG4B was found to be the most significantly differentially expressed gene (Figure 3B). Subsequently, a positive correlation was obtained between YY1 and ATG4B expression (*n* = 375, r = 0.72, *P* = 2.31e-61) in GC patient data from the UCSC Xena platform (Figure 3C).

Studies have revealed that ATG4B promotes autophagy, and the elevated expression of ATG4B is associated with advanced metastasis and poor prognosis in GC [31]. Additionally, ATG4B-targeted inhibition results in reduced autophagy flux and tumorigenicity in glioblastoma cells [32]. The expression of ATG4B in GC cells was assessed using RT-qPCR, resulting in significantly highly expressed ATG4B in cancer cells than in normal GES-1 cells (Figure 3D). The ChIP (chromatin immunoprecipitation) assay established binding between YY1 and the ATG4B promoter (Figure 3E). Next, the specific DNA binding sequence of YY1 on the ATG4B promoter (AAAATGGCGGCTCCC, -135/-121 bases from its transcription start site) obtained from the UCSC database (http://genome.ucsc.edu/) was identified to design the mutated reporter of ATG4B promoter (Figure 3F). Notably, the luciferase activity of the mutated ATG4B promoter was not affected by YY1 in GC cells (Figure 3G). Then, stable activation or inhibition of YY1 using dCas9 applications facilitated or attenuated the promoter activity, mRNA transcripts and protein expression levels of ATG4B in MG803 and AGS cells (Figure 3G–3I). These results indicated that YY1 facilitated the transcription of ATG4B to promote autophagy in cancer.

### ALKBH5 weakens the m6A levels of YY1 mRNA

**Figure 4:**
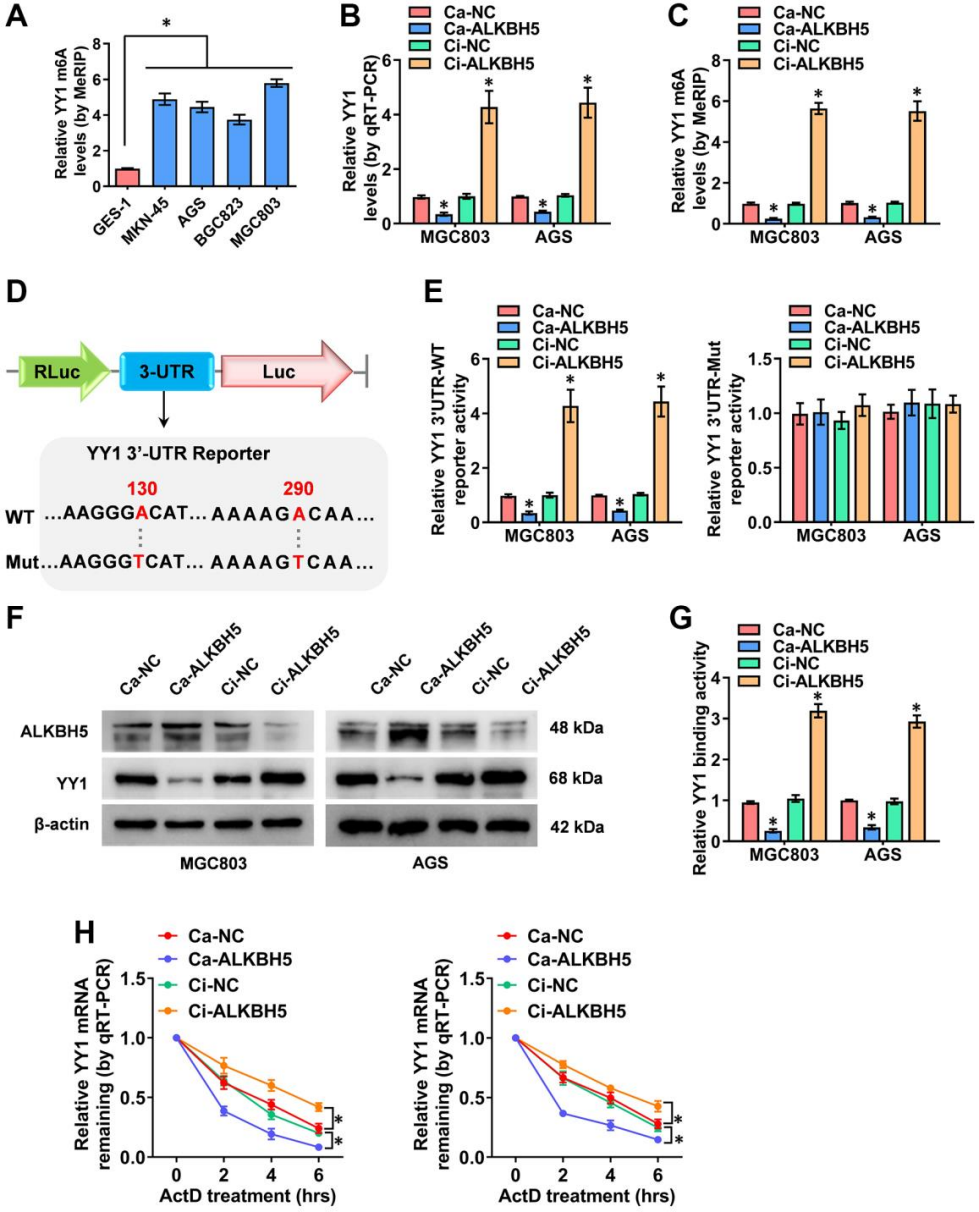
**ALKBH5 weakens the m6A levels of YY1 mRNA.** (**A**) MeRIP assay showing the YY1 m6A levels in human GC cell lines compared to GES-1 cells. (**B**, **C**) qRT-PCR and MeRIP assays displaying the mRNA and m6A levels in MGC803 and AGS cells induced for the overexpression or knockdown of ALKBH5. (**D**) Schematic illustration showing the m6A modification site (AAGGGA, AAAAGA) at position of 130 and 290 base on the YY1 3’-UTR from SRAMP Browser, and the wild-type (WT) or mutation (Mut) of YY1 3’-UTR reporters were designed (m6A was replaced by T). (**E**, **F**) Dual-luciferase and Western blot assays revealing the 3’-UTR activity and protein levels of YY1 in GC cells stably transfected with Ca-NC, Ca-ALKBH5, Ci-NC or Ci-ALKBH5. (**G**, **H**) Dual-luciferase and qRT-PCR assays showing the binding activity and mRNA half-life of YY1 in GC cells stably transfected with Ca-NC, Ca-ALKBH5, Ci-NC or Ci-ALKBH5. ^*^P < 0.05 vs. GES-1, Ca-NC, Ci-NC.

According to previous studies, m6A modifications are the most prevalent internal regulations on eukaryotic mRNAs, playing key roles in cancer progression [21]. Critically, the m6A methyltransferase of METTL3 enhances YY1 mRNA stability and tumorigenesis in an m6A-dependent manner [28]. Nevertheless, the m6A regulation mechanisms of YY1 and its m6A regulator in GC are rarely reported. Herein, to probe the outcome of the m6A modification of YY1, the MeRIP-qPCR (methylated RNA immunoprecipitation-qPCR) assay was deployed, revealing that the m6A levels of YY1 were significantly higher in GC cells than in normal GES-1 cells (Figure 4A). Particularly, the m6A eraser that induced YY1 mRNA demethylation was not clear, and ALKBH5 (alkB homolog 5, RNA demethylase) and FTO (FTO alpha-ketoglutarate dependent dioxygenase) were the most reported and strongest m6A demethylases. As a result, the regulatory functions of these two m6A regulators on YY1 were detected. Notably, the overexpression or knockdown of ALKBH5 decreased or increased the mRNA and m6A levels of YY1 according to RT-qPCR and MeRIP-qPCR assays, respectively, but there were no changes in the FTO groups (Figure 4B, 4C and Supplementary Figure 3A, 3B). As the m6A modifications are mainly enriched in the 3′-UTR near the mRNA stop codon [33], and the SRAMP Browser (http://www.cuilab.cn/sramp/) revealed the m6A modification site with very high confidence on the YY1 3′-UTR, the wild-type or mutations of YY1 3′-UTR reporters were designed (Figure 4D). Per dual-luciferase and western blot assays, the stable activation or inhibition of ALKBH5 respectively attenuated or facilitated the 3′-UTR activity, protein expression levels and binding activity of YY1 in MGC803 and AGS cells (Figure 4E–4G). Notably, the luciferase activity of the mutated YY1 3′-UTR was not affected by ALKBH5 in GC cells (Figure 4E). To examine the regulation of mRNA stability, the RNA polymerase II inhibitor of actinomycin D was used, with the results revealing that ALKBH5 reduced the mRNA half-life of YY1 in GC cells (Figure 4H). In summary, ALKBH5 altered the stability of YY1 mRNA by reducing its m6A levels, leading to YY1 inhibition in GC cells.

### The M6A-dependent promotion of YY1 is positively associated with YTHDF1

**Figure 5:**
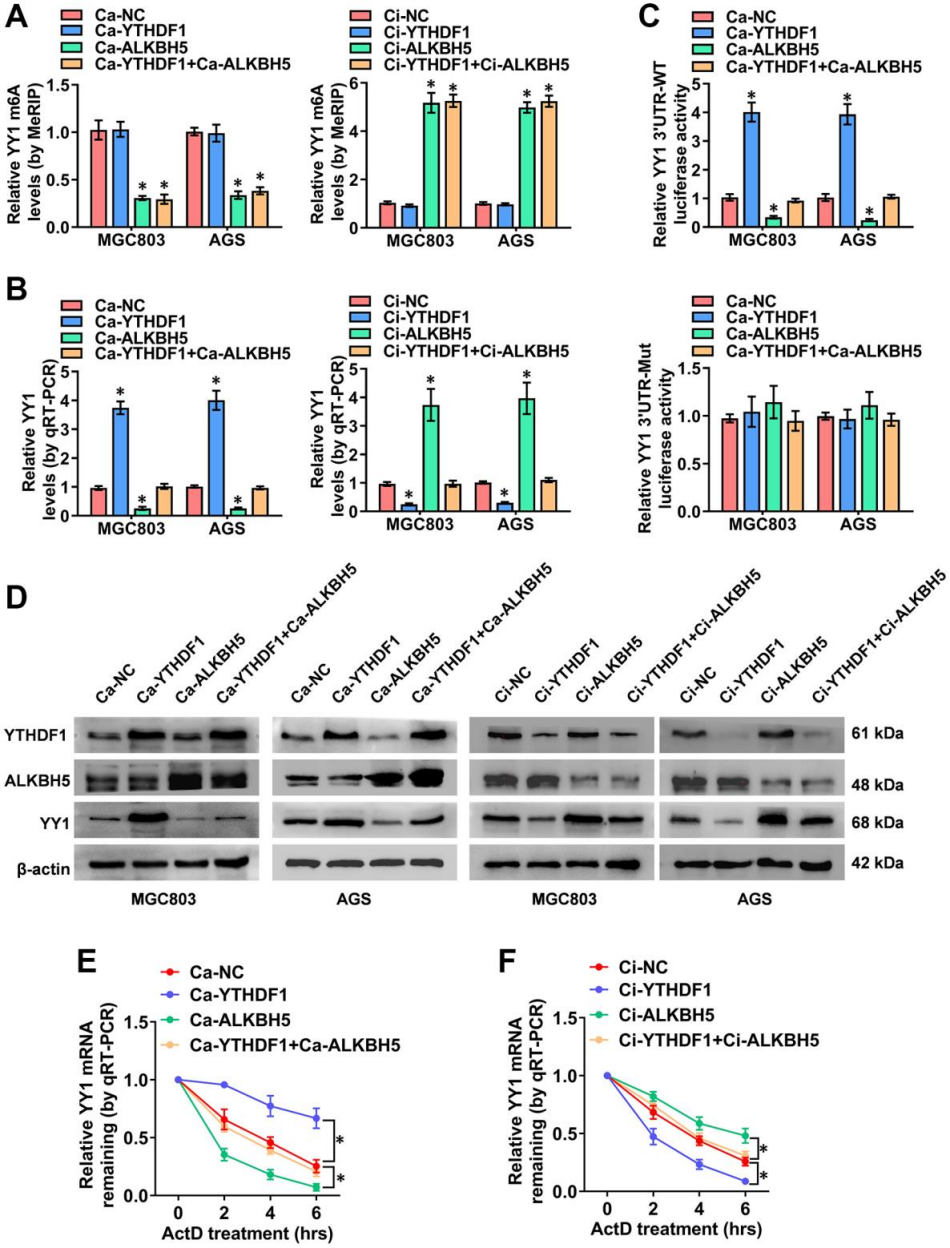
**The m6A-dependent promotion of YY1 is positively associated with YTHDF1.** (**A**–**D**) MeRIP, qRT-PCR, dual luciferase and western blot assays displaying the m6A and mRNA levels, as well as the 3'-UTR activity and protein expression of YY1 in MGC803 and AGS cells transfected with Ca-YTHDF1 or Ci-YTHDF1 and co-transfected with Ca-ALKBH5 or Ci-ALKBH5. (**E**, **F**) qRT-PCR assay showing the half-life levels of YY1 mRNA in AGS cells treated with actinomycin D (1 μg/ml) at the indicated periods. ^*^P < 0.05 vs. Ca-NC, Ci-NC.

While ALKBH5-mediated mRNA demethylation suppresses YY1 expression, the specific m6A reader that recognizes this m6A-modified YY1 mRNA and affects its function is yet unidentified. It is widely accepted that m6A modification effects are mediated via interactions between m6A sites and m6A reader proteins, including YTHDF1-3, IGF2BP1-3 and YTHDC1-2 [34], with YTHDF1 reportedly promoting YY1 mRNA and protein levels through binding to its m6A-methylated transcripts [35]. Importantly, previous study [36] has identified YTHDF1 as a potential m6A reader on the YY1 3′-UTR in an iCLIP (individual-nucleotide resolution UV crosslinking and immunoprecipitation) data (GSE78030). The YTHDF1 binding region analyzed from the m6A-Atlas v2.0 platform (http://rnamd.org/m6a/index.php, source data: GSE78030) is from chr14:100277763 to 100277903. Obviously, the enrichment peak region and binding sites of YTHDF1 on YY1 mRNA reveled from SRAMP and m6A-Atlas database are highly consistent. To further clarify this specific potential molecular mechanism, the YTHDF1-regulated YY1 m6A levels and expression features were assessed. MeRIP-qPCR experiments confirmed that YTHDF1 activation and its interference with the dCas9 methods (Ca and Ci) had no impact on YY1 m6A levels; however, ALKBH5 regulated YY1 m6A levels (Figure 5A). Notably, as the RT-qPCR dual-luciferase and western blot assay results show in Figure 5B, 5D, the overexpression and knockdown of YTHDF1 promoted and suppressed, respectively, the mRNA levels, 3′-UTR activity and protein expression of YY1; however, these effects were rescued after co-transfection with Ca-ALKBH5 or Ci-ALKBH5. Notably, the luciferase activity of the mutated YY1 3′-UTR was not affected by ALKBH5 and YTHDF1 in GC cells (Figure 5C and Supplementary Figure 3C). The effects of YTHDF1 on YY1 mRNA stability were also examined. As shown in Figure 5E, the stable overexpression of ALKBH5 attenuated the YTHDF1-induced increase in the half-life of YY1 mRNA, while the stable knockdown of ALKBH5 prolonged the reduced degradation time of YY1 mRNA in AGS cells through YTHDF1 inhibition (Figure 5F). Overall, these results suggest that the m6A-methylated YY1 mRNA was directly identified and stabilized by YTHDF1, and YY1 was regulated in GC via the m6A-ALKBH5-YTHDF1 axis.

### YY1 drives cancer progression and autophagy in an m6A-dependent manner

**Figure 6:**
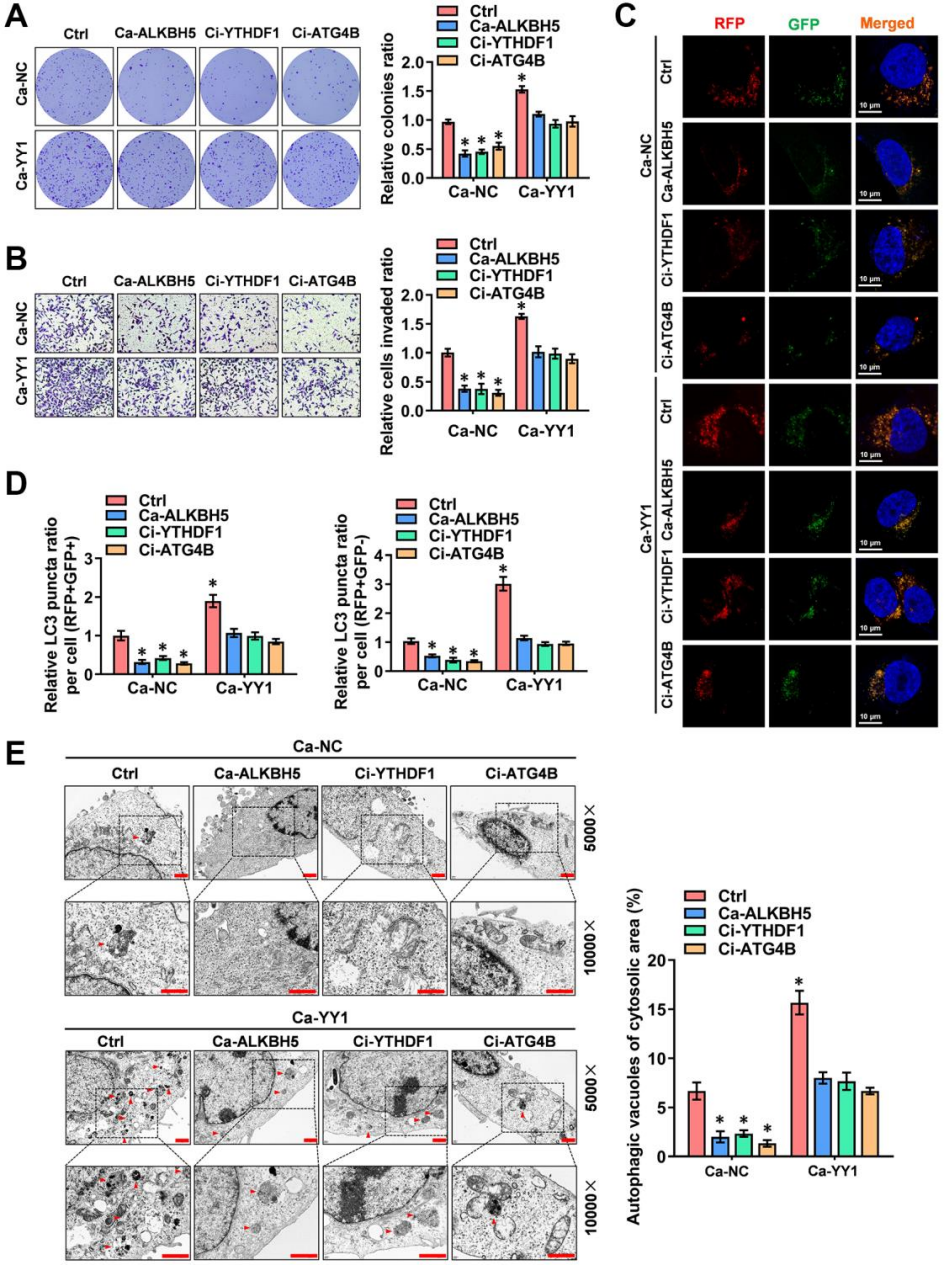
**YY1 drives cancer progression and autophagy in an m6A-dependent manner.** (**A**, **B**) Representative images (left) and the quantification (right) of colony formation (**A**) and transwell (**B**) assays showing the growth and migration of AGS cells transfected with Ca-NC, Ca-YY1 or co-transfected with Ca-ALKBH5, Ci-YTHDF1 or Ci-ATG4B. (**C**, **D**) Representative images (**C**) and quantification (**D**) revealing the immunofluorescence staining intensity with mRFP-GFP-LC3 in AGS cells stably transfected with Ca-NC, Ca-YY1 or co-transfected with Ca-ALKBH5, Ci-YTHDF1 or Ci-ATG4B. Red (RFP+GFP-) puncta represent autolysosomes, and yellow (RFP+GFP+) puncta represent autophagosomes. Scale bar: 10 μm. (**E**) Representative images from TEM scanning (left panel) and quantification (right panel) exhibiting autophagic vacuoles in AGS cells stably transfected with Ca-NC, Ca-YY1 or co-transfected with Ca-ALKBH5, Ci-YTHDF1 or Ci-ATG4B. Scale bar: 1 μm. ^*^P < 0.05 vs. Ca-NC+Ctrl.

To further reinforce the findings here, a rescue assay was performed to detect the role of YY1 in regulating the autophagic pathway and cancer progression in GC cells. YY1 overexpression facilitated the growth and migration capacity of MGC803 and AGS cells; however, these activities were rescued by ALKBH5 activation or YTHDF1 and ATG4B inhibition (Figure 6A, 6B and Supplementary Figure 4A, 4B). Next, the regulatory effects of m6A-YY1-ATG4B on autophagy in GC cells were tested. As shown in the results, YY1 enhanced the expression of ATG4B (Supplementary Figure 4C) and LC3B associated with the decreased expression of P62 in MGC-803 and AGS cells, while ALKBH5 activation or YTHDF1 and ATG4B inhibition abolished these effects (Supplementary Figure 4D). The autophagy flux assay and TEM analysis findings showed that the overexpression of YY1 accelerated the autophagosome and autolysosome formation of AGS cells, which were rescued by ALKBH5 activation or YTHDF1 and ATG4B inhibition (Figure 6C–6E). Taken together, these results suggest that YY1 promoted autophagy and GC progression in an m6A dependent manner.

### The inhibition of YY1 suppresses tumorigenesis *in vivo*

**Figure 7:**
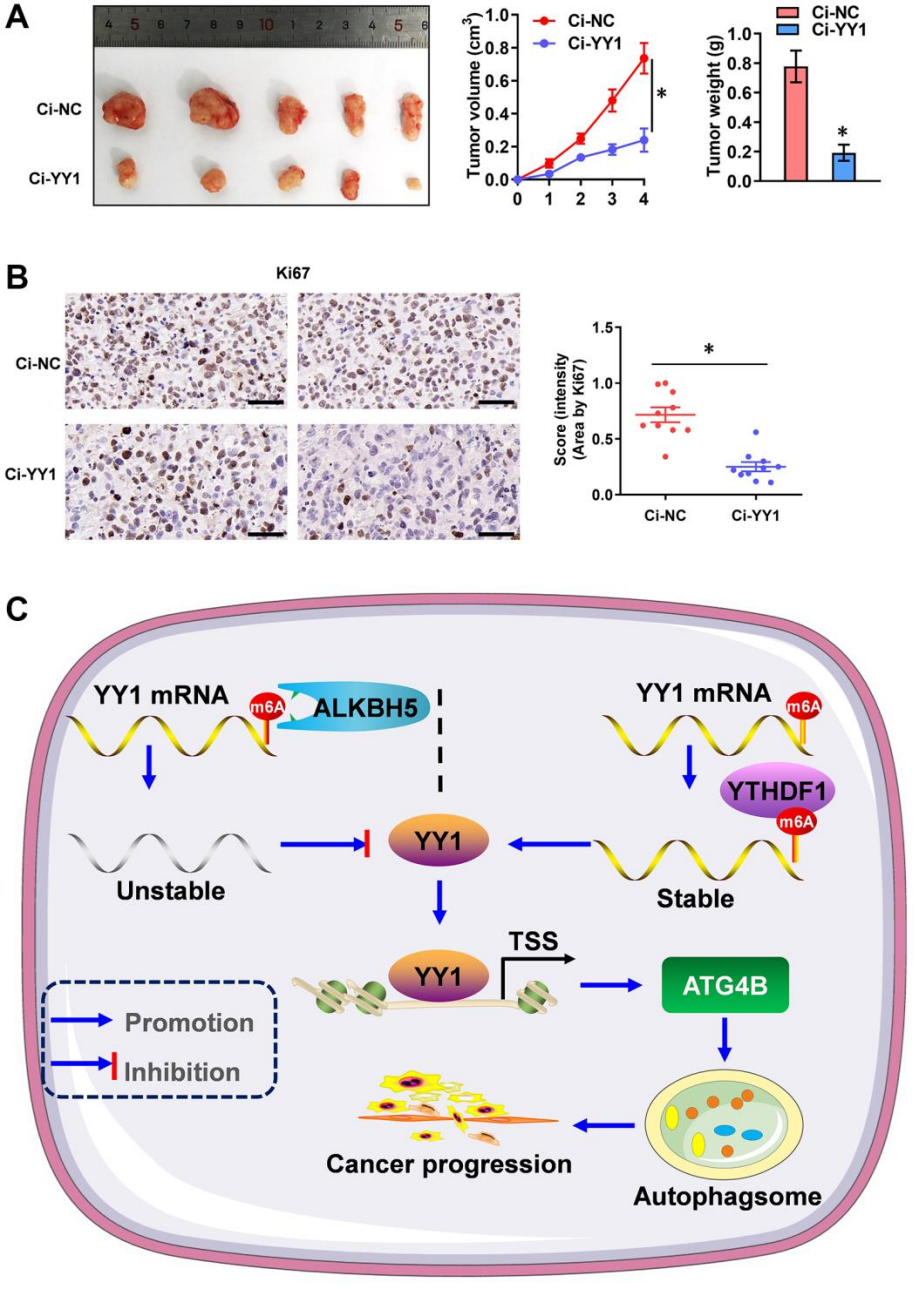
**YY1 inhibition suppresses tumorigenesis *in vivo*.** (**A**) Representative images, *in vivo* growth curve, and tumor weights of xenografts in nude mice established by the subcutaneous injection of AGS cells stably transfected with Ci-NC, Ci-YY1. (**B**) Representative images and IHC quantification staining showing Ki-67 protein expression in xenograft tumors. (**C**) Mechanisms underlying YY1-promoted autophagy and GC progression. ^*^P < 0.05 vs. Ci-NC.

Additionally, the therapeutic values of YY1 inhibition were explored via dCas9 application on tumor growth *in vivo*. The stable knockdown of YY1 suppressed tumor growth and weight, as well as the Ki-67 expression (marker of proliferation) of xenografts in AGS cell-engineered athymic nude mice (Figure 7A, 7B), suggesting that the inhibition of YY1 suppresses tumorigenesis *in vivo*. Overall, these results point to YY1 being regulated by ALKBH5-mediated m6A modifications. They also suggest that YY1 promoted autophagy and GC progression through targeting ATG4B (Figure 7C).

## DISCUSSION

The role of cellular autophagy in tumor progression is essential and intricate, changing with tumor types and tumor stages. Therefore, valuable theoretical significance and clinical application prospects can be drawn from elucidating the role of autophagy. Studies have shown that autophagy guards GC cells against vincristine-induced apoptosis [37], and activated carcinogenic autophagy facilitates GC progression [38]. Autophagy inhibition can sensitize drug-resistant cancer cells and enhance the antitumor effects of chemotherapeutic drugs. For instance, silencing ATG5 re-sensitizes drug-resistant cells to chemotherapy again [39]. ATG4B reportedly plays a pivotal role in autophagy during the processing of the LC3 protein, cleaving the newly synthesized LC3 at the initial phase of autophagy [40]. One investigation found ATG4B activity to be essential to HDAC4-induced autophagic MEKK3 degradation in GC [41]. Currently, a great deal of effort is being devoted to elucidating the function and downstream mechanisms of autophagy [42–44], but very few studies are focusing on the upstream regulatory mechanisms of autophagy.

YY1, a highly conserved transcription factor, plays essential roles in the regulation of a range of genes [45]. It can recruit the acetyltransferase and deacetylase of histone to the promoter of target genes, activating or inhibiting gene expression [46]. YY1 also promotes PVT1 expression via binding to its promoter to further affect autophagy through the mTOR pathway, leading to increased invasion and adhesion activity in cancer [47]. Although accumulating evidence suggests that YY1 has a promotional role in tumors, studies have recently demonstrated otherwise. Additionally, YY1 has been shown to inhibit pancreatic ductal adenocarcinoma progression by suppressing MMP2 expression [48]. Furthermore, it regulates BRCA1 expression in breast cancer cells and ultimately inhibits tumor growth [49]. Thus, as a multifunctional regulator possessing significant biological functions, YY1 has the potential to be a key regulator of cancer development. In the present study, YY1 acted as a key regulator of the autophagy machinery genes in GC. YY1 also promoted the proliferation and migration of GC cells, and the autophagy inhibitor of 3-MA incubation inhibited the effect of YY1 on these cells. Mechanistically, YY1 activated the ATG4B-dependent autophagic pathway through binding to the promoter of ATG4B, leading to the enhancement of autophagy and GC progression. YY1-mediated autophagy stimulation is, therefore, a potential target for the treatment of GC.

Epigenetics engages and regulates the flow of information from DNA to RNA to protein at multiple levels, with emerging studies finding epigenetic modifications of RNA crucial to biological processes [44]. In eukaryotes, more than 100 RNA modifications have been found along with Cap at the 5′ end and ploy-A modification at the 3′ end, playing vital roles in transcriptional regulation. M6A is the most common internal modifier of mRNA, with a large number of enzymes involved with m6A having been identified [50]. M6A-modified mRNAs are connected to the terminal fate of tumors, and targeting key regulators of m6A methylation modifications to promote anti-cancer therapy is a promising prospect. However, the exploration of the potential mechanisms of m6A modification in GC is sparse. As demonstrated in this study, the YY1 m6A levels were significantly elevated in GC cells, and the m6A-ALKBH5-YTHDF1 axis impacted the stability of YY1 mRNA. Notably, ALKBH5 reduced YY1 methylation, while YTHDF1 promoted its m6A methylation recognition sensitivity and mRNA stabilization and, eventually, YY1 expression.

In summary, the current probe showed that YY1 was drastically up-regulated in GC, and the ectopic expression of YY1 induced ATG4B-dependent autophagy, enhancing the proliferation and metastasis activity of GC cells. The data obtained also revealed that ALKBH5, thanks to its ability to halt the methylation of YY1 mRNA, negatively regulated YY1 expression. Furthermore, YTHDF1 recognized YY1 mRNA methylation modifications to maintain its stabilization, resulting in increased YY1 expression. Hence, these results point to the M6A/YY1/ATG4B axis being a potential therapeutic approach for GC.

## Supplementary Materials

Supplementary Figures

Supplementary Tables
