# Supplementary material for: YY1 is regulated by ALKBH5-mediated m6A modification and promotes autophagy and cancer progression through targeting ATG4B
Source: Aging (Albany NY). 2023 Sep 18;15(18):9590–613. doi: 10.18632/aging.205037 (PMC10564435; doi:10.18632/aging.205037)
Supplement: Supplementary Figures [file aging-15-205037-s001.pdf]

SUPPLEMENTARY FIGURES

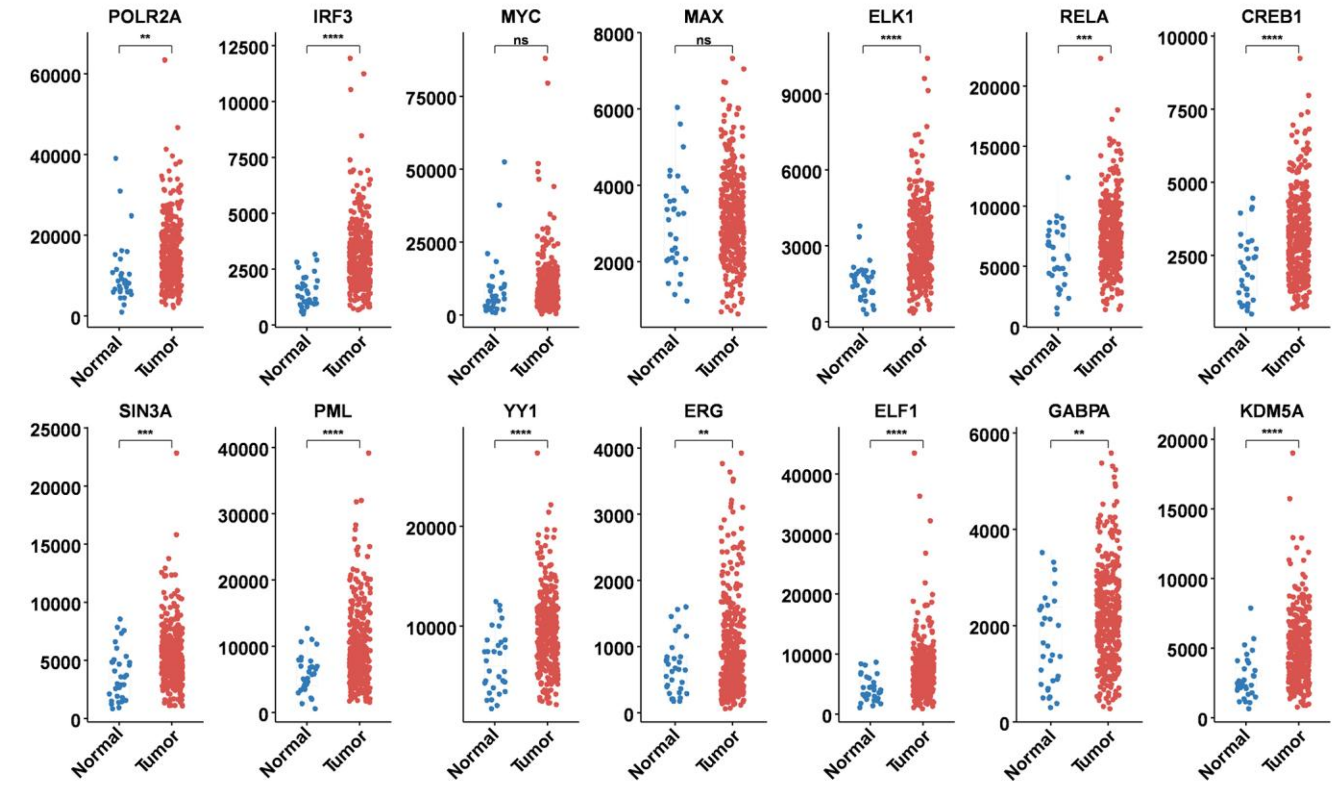

Supplementary Figure 1. Expression profiles of 14 transcription factors related in autophagy from TCGA database.

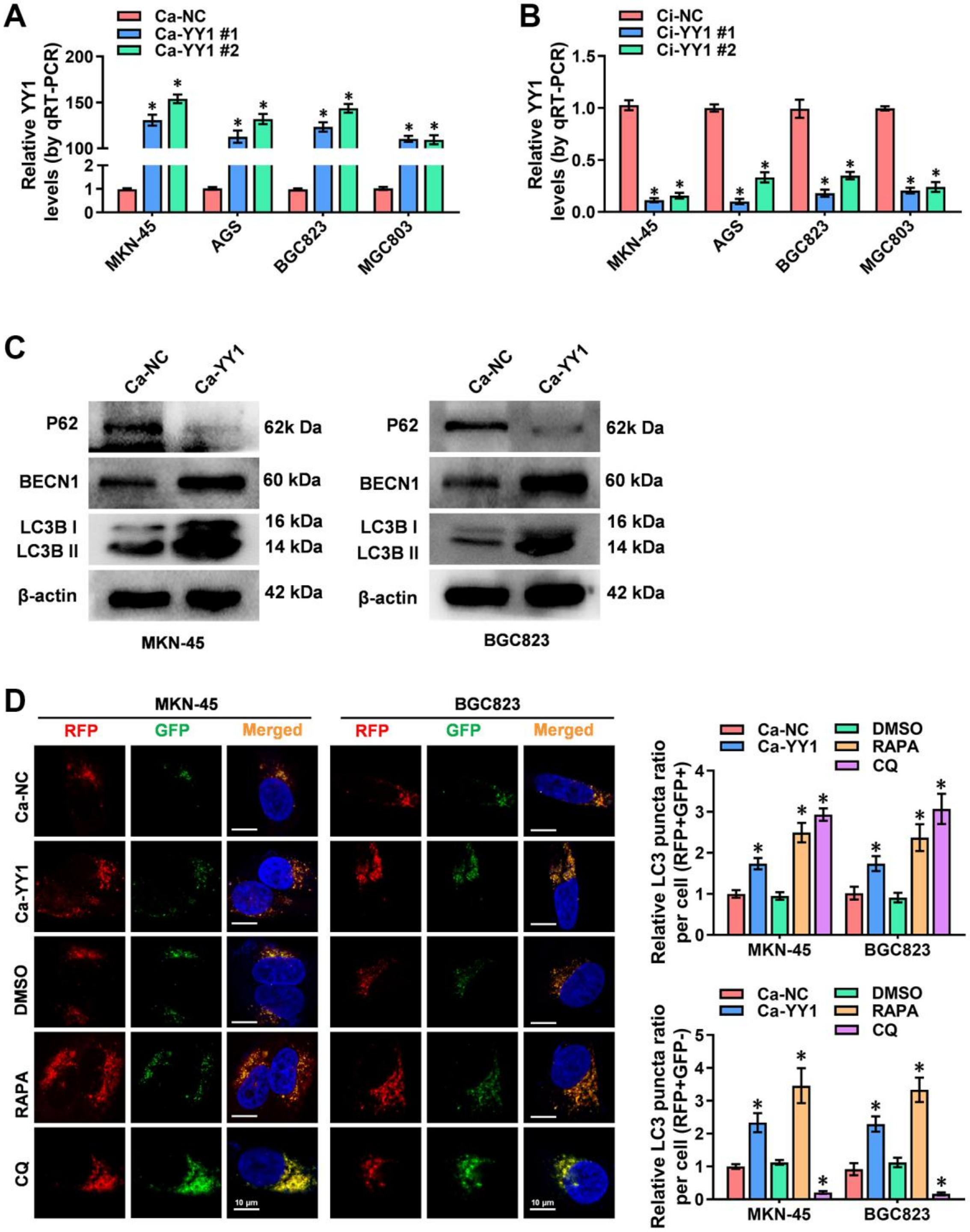

Supplementary Figure 2. YY1 promotes autophagy in cancer. (**A**, **B**) Real - time qRT - PCR analysis verifying the effective activation or repression in MGC803, AGS, MKN-45 and BGC823 cells transfected with Ca-NC, Ca-YY1 #1, #2, or Ci-NC, Ci-YY1 #1, #2. (**C**) Western blotting showing the expression of the autophagy-associated proteins of LC3B, p62, and BECN1 in YY1-overexpressing or YY1 knockdown cells. (**D**) Representative images (left panel) and quantification (right panel) showing the immunofluorescence staining intensity with mRFP-GFP-LC3 in MKN-45 and BGC823 cells treated with the empty vector (Ca-NC) or Ca-YY1, and those treated with DMSO, CQ (Chloroquine, 20 μmol/L) or RAPA (Rapamycin, 1 μmol/L) as positive controls for inactivated or activated autophagy. Scale bar: 10 μm. $^{*}P < 0.05$ vs. Ca-NC, Ci-NC, DMSO.

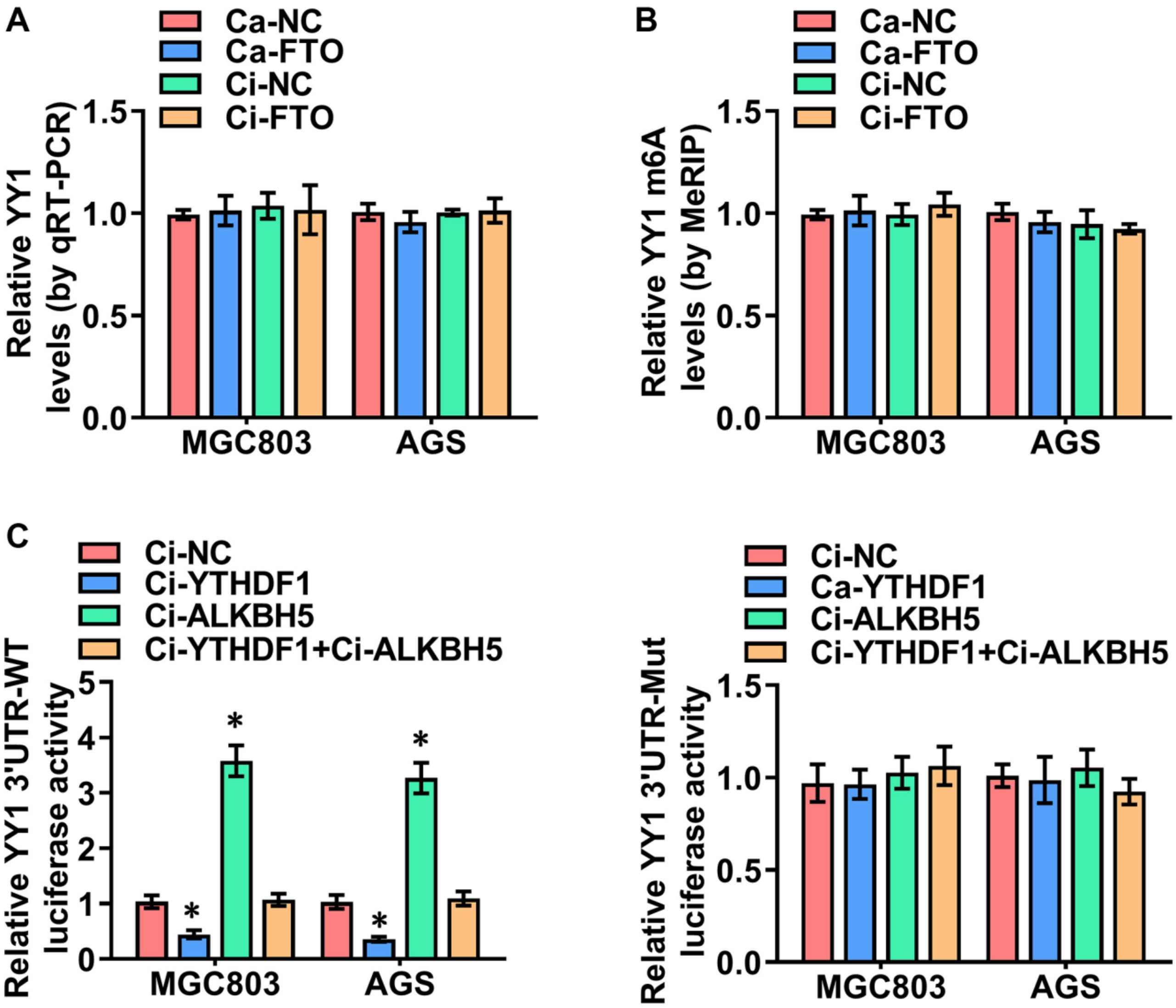

Supplementary Figure 3. YY1 was regulated via m6A-ALKBH5-YTHDF1 axis. (**A**, **B**) qRT-PCR and MeRIP assays displaying the mRNA and m6A levels in MGC803 and AGS cells induced for the overexpression or knockdown of FTO. (**C**) dual luciferase assay displaying the 3'-UTR activity of YY1 in MGC803 and AGS cells transfected with Ci-YTHDF1 and co-transfected with Ci-ALKBH5. $^{*}P < 0.05$ vs. Ca-NC, Ci-NC.

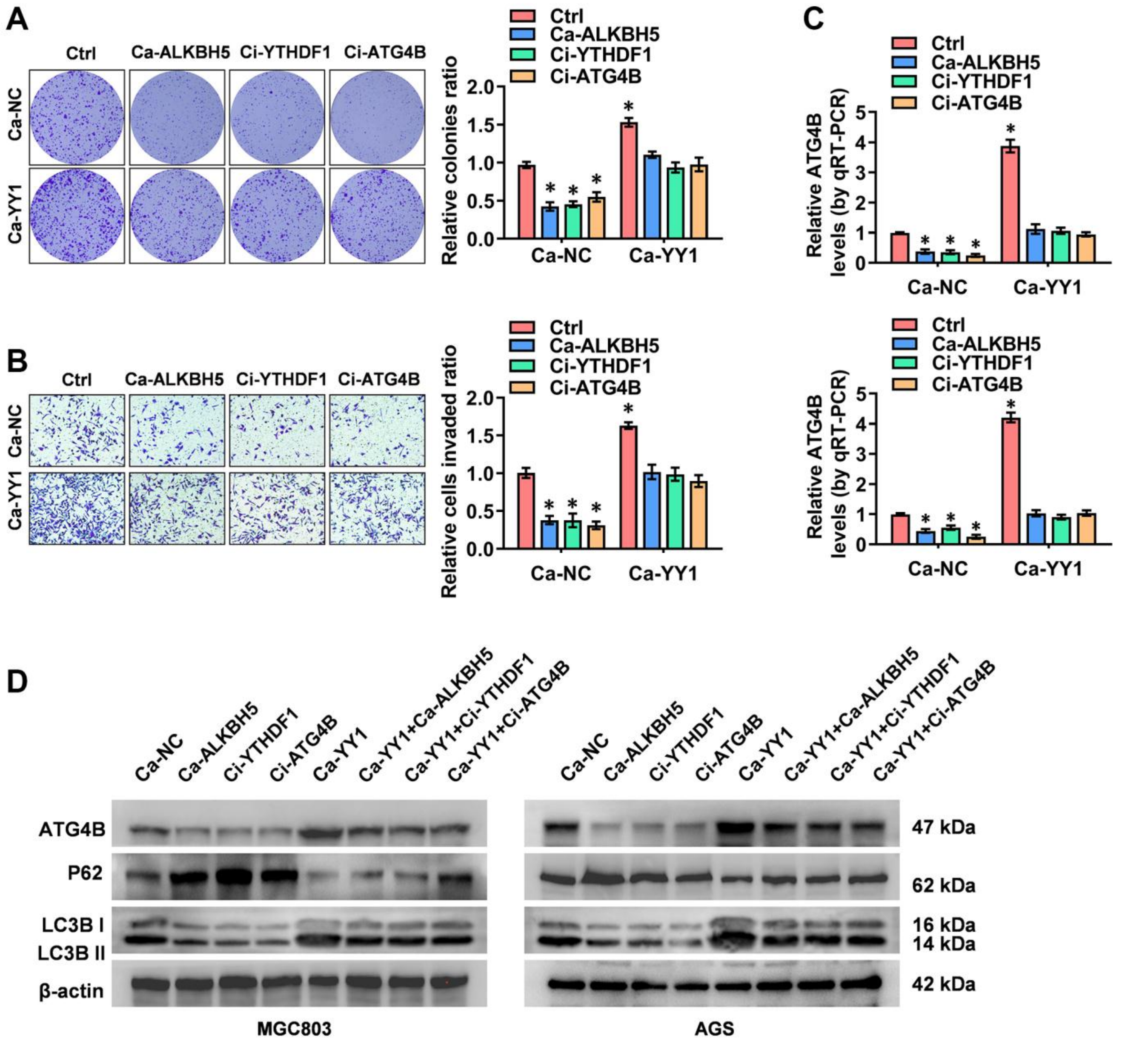

Supplementary Figure 4. YY1 drives cancer progression and autophagy in an m6A-dependent manner. (**A**, **B**) Representative images (left) and the quantification (right) of colony formation (**A**) and transwell (**B**) assays showing the growth and migration of MGC803 cells transfected with Ca-NC, Ca-YY1 or co-transfected with Ca-ALKBH5, Ci-YTHDF1 or Ci-ATG4B. (**C**) Real - time qRT - PCR assay indicating the levels of ATG4B in MGC803 and AGS cells transfected with Ca-YY1, or co-transfected with Ca-ALKBH5, Ci-YTHDF1 or Ci-ATG4B. (**D**) Western blotting showing the expression of the autophagy-associated proteins of LC3B and p62 in MGC803 and AGS cells transfected with Ca-YY1, or co-transfected with Ca-ALKBH5, Ci-YTHDF1 or Ci-ATG4B. *$P < 0.05$ vs. Ca-NC+Ctrl.
