# Supplementary material for: YY1 is regulated by ALKBH5-mediated m6A modification and promotes autophagy and cancer progression through targeting ATG4B
Source: Aging (Albany NY). 2023 Sep 18;15(18):9590–613. doi: 10.18632/aging.205037 (PMC10564435; doi:10.18632/aging.205037)
Supplement: Supplementary Tables [file aging-15-205037-s002.pdf]

## SUPPLEMENTARY TABLES

### Supplementary Table 1. Primer sets used for qPCR, RT-PCR.

| Primer set 1 | Primers | Sequence | Product size (bp) |
|---|---|---|---|
| ACTB | Forward | 5′-TGCCCATCTACGAGGGGTATG-3′ | 156 |
| | Reverse | 5′-TCTCCTTAATGTCACGCACGATTT-3′ | |
| ALKBH5 | Forward | 5′-GTTCAAGCCTATTCGGGTGT-3′ | 204 |
| | Reverse | 5′-ACGGAGCTGCTCAGGGACT-3′ | |
| ATG4B | Forward | 5′-TCAGAGCCCGTTTGGATA-3′ | 283 |
| | Reverse | 5′-CGATGAATGCGTTGAGGAC-3′ | |
| GAPDH | Forward | 5′-AGAAGGCTGGGGCTCATTTG-3′ | 258 |
| | Reverse | 5′-AGGGGCCATCCACAGTCTTC-3′ | |
| YY1 | Forward | 5′-AAGTGGGAGCAGAAGCAGG-3′ | 297 |
| | Reverse | 5′-GCCTTTATGAGGGCAAGCT-3′ | |
| ATG4B (ChIP) | Forward | 5′-AGAGGAGGAAGCGCCACCCAT-3′ | 183 |
| | Reverse | 5′-CGCAGGCGGCGAAGACGATA-3′ | |
| YY1 (3′-UTR) | Forward | 5′-GAAGACCCTTCTCGACCACG-3′ | 356 |
| | Reverse | 5′-TAAGCAACAGGTGAGCTTCATA-3′ | |

### Supplementary Table 2. Oligonucleotide sets used for constructs.

| Oligo set | Sequences |
|---|---|
| Ci-YY1 #1 Target | 5′-ACCCTCTACATCGCCACGGACGG-3′; |
| Ci-YY1 #2 Target | 5′-GATGTAGAGGGTGTCGCCCGAGG-3′ |
| Ca-YY1 #1 Target | 5′-CGCTCGGCCGCTGCTCGTCTCGG-3′; |
| Ca-YY1 #2 Target | 5′-CGGGCCCGAGCAGAGTGTGGCGG-3′ |
| Ci- ALKBH5 #1 Target | 5′-AGCTCTCCGCACGACGTCACGGG-3′; |
| Ci- ALKBH5 #2 Target | 5′-TGGCTGCCCGTGACGTCGTGCGG-3′; |
| Ca- ALKBH5 #1 Target | 5′-GTTCGGACGATGCCGTGACGCGG-3′ |
| Ca- ALKBH5 #2 Target | 5′-ATATGAGCGCACCCCTGTAGAGG-3′ |
| Ci-ATG4B #1 Target | 5′-CCGGCCGTACGCCAAAATGGCGG-3′; |
| Ci-ATG4B #2 Target | 5′-GCGACGCCGCTCGGGTCAGTCGG-3′; |
| Ca-ATG4B #1 Target | 5′-GCGCGCGAGCGGAAATACGCGGG-3′ |
| Ca-ATG4B #2 Target | 5′-CAGCAACGCGACGCGGCGACGGG-3′; |
| Ci-FTO #1 Target | 5′-CTAAATCCCGTGGCGCTCGCGGG-3′; |
| Ci-FTO #2 Target | 5′-CGAGGGATCTACGCAGCTTGCGG-3′ |
| Ca-FTO #1 Target | 5′-CTATAGCGCCGACAGCGTGGCGG-3′ |
| Ca-FTO #2 Target | 5′-TATAGCGCCGACAGCGTGGCGGG-3′ |
| Ci-YTHDF1 #1 Target | 5′-TCCTCAGTGCGTCCGCGTCCCGG-3′; |
| Ci-YTHDF1 #2 Target | 5′-GGAGGCGTCGACTCCAATGGCGG-3′; |
| Ca-YTHDF1 #1 Target | 5′-GGTCCCAGTCTCGTGCGGCGGGG-3′ |
| Ca-YTHDF1 #2 Target | 5′-CGAAATCCATCCCGTAAGACGGG-3′ |
| pGL3-YY1 Reporter | 5′-CCGGCCATCTTGATACGACCATCTTCT ATCGGCCATCTTGATACGACCATGTTCA-3′ (sense); |
| | 5′-AGCTTGAACATGGTCGTATCAAGATGGCCGAT AGAAGATGGTCGTATCAAGATGGCCGGGTAC-3′(antisense) |

| | |
|---|---|
| pGL3-ATG4B promoter | 5′-GCCGCTCGAGCCGATGTCCTTTCCTCCCATACCA-3′ (sense)<br>5′-CCCAAGCTTCTCCTCACCTGCGTCCATCTTCC-3′ (antisense) |

**Supplementary Table 3. Screening for transcription factors in autophagy.**

| Autophagy related genes | | | | | | | |
|---|---|---|---|---|---|---|---|
| AMBRA1 | BCL2L1 | CHMP2B | FADD | IKBKE | MYC | PTEN | TBK1 |
| APOL1 | BECN1 | CHMP4B | FAM48A | IL24 | NAF1 | PTK6 | TM9SF1 |
| ARNT | BID | CLN3 | FAS | IRGM | NAMPT | RAB11A | TMEM49 |
| ARSA | BIRC5 | CTSB | FKBP1A | ITGA3 | NBR1 | RAB1A | TMEM74 |
| ARSB | BIRC6 | CTSD | FKBP1B | ITGA6 | NCKAP1 | RAB24 | TNFSF10 |
| ATF4 | BNIP1 | CTSL1 | FOS | ITGB1 | NFE2L2 | RAB33B | TP53 |
| ATF6 | BNIP3 | CX3CL1 | FOXO1 | ITGB4 | NFKB1 | RAB5A | TP53INP2 |
| ATG10 | BNIP3L | CXCR4 | FOXO3 | ITPR1 | NKX2-3 | RAB7A | TP63 |
| ATG12 | C12orf44 | DAPK1 | GAA | KIAA0226 | NLRC4 | RAC1 | TP73 |
| ATG16L1 | C17orf88 | DAPK2 | GABARAP | KIAA0652 | NPC1 | RAF1 | TSC1 |
| ATG16L2 | CALCOCO2 | DDIT3 | GABARAPL1 | KIAA0831 | NRG1 | RB1 | TSC2 |
| ATG2A | CAMKK2 | DIRAS3 | GABARAPL2 | KIF5B | NRG2 | RB1CC1 | TUSC1 |
| ATG2B | CANX | DLC1 | GAPDH | KLHL24 | NRG3 | RELA | ULK1 |
| ATG3 | CAPN1 | DNAJB1 | GNAI3 | LAMP1 | P4HB | RGS19 | ULK2 |
| ATG4A | CAPN10 | DNAJB9 | GNB2L1 | LAMP2 | PARK2 | RHEB | ULK3 |
| ATG4B | CAPN2 | DRAM1 | GOPC | MAP1LC3A | PARP1 | RPS6KB1 | USP10 |
| ATG4C | CAPNS1 | EDEM1 | GRID1 | MAP1LC3B | PEA15 | RPTOR | UVRAG |
| ATG4D | CASP1 | EEF2 | GRID2 | MAP1LC3C | PELP1 | SAR1A | VAMP3 |
| ATG5 | CASP3 | EEF2K | HDAC1 | MAP2K7 | PEX14 | SERPINA1 | VAMP7 |
| ATG7 | CASP4 | EGFR | HDAC6 | MAPK1 | PEX3 | SESN2 | VEGFA |
| ATG9A | CASP8 | EIF2AK2 | HGS | MAPK3 | PIK3C3 | SH3GLB1 | WDFY3 |
| ATG9B | CCL2 | EIF2AK3 | HIF1A | MAPK8 | PIK3R4 | SIRT1 | WDR45 |
| ATIC | CCR2 | EIF2S1 | HSP90AB1 | MAPK8IP1 | PINK1 | SIRT2 | WDR45L |
| BAG1 | CD46 | EIF4EBP1 | HSPA5 | MAPK9 | PPP1R15A | SPHK1 | WIPI1 |
| BAG3 | CDKN1A | EIF4G1 | HSPA8 | MBTPS2 | PRKAB1 | SPNS1 | WIPI2 |
| BAK1 | CDKN1B | ERBB2 | HSPB8 | MLST8 | PRKAR1A | SQSTM1 | ZFYVE1 |
| BAX | CDKN2A | ERN1 | IFNG | MTMR14 | PRKCD | ST13 | |
| BCL2 | CFLAR | ERO1L | IKBKB | MTOR | PRKCQ | STK11 | |

| Transcription factors analysis from rcis rarget | | | | | | | |
|---|---|---|---|---|---|---|---|
| ARID3A | BHLHE40 | ELF2 | FEV | KDM5A | PITX3 | SREBF1 | ZFP42 |
| ARNT | BHLHE41 | ELF4 | FLI1 | MAFK | PML | SREBF2 | ZKSCAN3 |
| ARNT2 | BPTF | ELK1 | FOXD1 | MAX | POLR2A | TEAD4 | ZNF358 |
| ARNTL | CEBPZ | ELK3 | FOXD2 | MITF | POU5F1 | TFE3 | ZNF362 |
| ARNTL2 | CREB1 | EP300 | FOXO3 | MLX | RAD21 | TFEB | ZNF384 |
| ATF2 | CREB3 | ERG | FOXP1 | MLXIPL | RELA | TFEC | ZNF454 |
| ATF3 | CREB3L1 | ETV2 | GABPA | MYC | SIN3A | USF1 | ZNF513 |
| ATF4 | CREB3L2 | ETV4 | GTF2F1 | NFE2 | SP4 | USF2 | ZNF597 |
| BACH1 | CREB3L4 | ETV4 | HELT | PAX4 | SPI1 | XBP1 | ZNF768 |
| BACH2 | CREM | ETV6 | IRF3 | PITX1 | SPIB | YY1 | |
| BATF3 | ELF1 | ETV7 | JDP2 | PITX2 | SPIC | YY2 | |

| Transcription factors analysis BART | | | | | | | |
|---|---|---|---|---|---|---|---|
| AFF4 | CDK8 | ELL2 | HSF1 | MAX | PAF1 | SAP30 | TBP |
| ATF1 | CDK9 | EMSY | INO80 | MAZ | PHF2 | SIN3A | TFDP1 |
| AUTS2 | CHD1 | EPC1 | IRF1 | MED1 | PHF8 | SIX5 | U2AF1 |
| BCL11B | CHD4 | ERG | IRF3 | MLLT1 | PHIP | SMAD5 | UBTF |
| BCL3 | CPSF3L | ETS1 | KAT5 | MYC | PML | SP1 | XRN2 |
| BRCA1 | CREB1 | FAM208A | KAT7 | MYCN | POLR2A | SP2 | YY1 |
| BRD1 | DCP1A | FOXM1 | KDM2B | NCOR1 | POLR2B | SPIN1 | ZBTB11 |
| BRD2 | DDX21 | GABPA | KDM5A | NELFA | RB1 | SRSF3 | ZBTB33 |
| BRD3 | E2F1 | GLI2 | KDM5C | NELFE | RBBP5 | STAT1 | ZBTB7A |
| BRD4 | E2F4 | GMEB2 | KLF15 | NFATC1 | RBL2 | STAT2 | ZNF143 |
| BRPF3 | EGR1 | GTF2B | KLF9 | NFYA | RBP2 | SUPT5H | ZSCAN22 |
| CBFB | ELF1 | H2AZ | KMT2A | NR2C2 | REL | TAF1 | |
| CD74 | ELK1 | HCFC1 | KMT2D | NRF1 | RELA | TAF3 | |
| CDK7 | ELK4 | HDAC1 | MAPK1 | OGT | RPE | TAFII | |

| Overlap | | | | | | | |
|---|---|---|---|---|---|---|---|
| CREB1 | ELF1 | ELK1 | ERG | GABPA | IRF3 | KDM5A | MAX |
| MYC | PML | POLR2A | RELA | SIN3A | YY1 | | |

**Supplementary Table 4. Screening for YY1 target autophagy related genes (Figure 3A).**

| YY1 target autophagy related genes | | YY1 related gene | Overlap |
|---|---|---|---|
| ARNT | MAPK9 | 1044 genes | ARNT |
| ATF4 | NRG1 | (Not shown) | ATG4B |
| ATG4B | PRKAR1A | | GOPC |
| BAX | RAB1A | | RAF1 |
| CDKN1B | RAB5A | | |
| CDKN2A | RAB7A | | |
| FKBP1B | RAF1 | | |
| FOXO1 | RHEB | | |
| GAPDH | RPTOR | | |
| GOPC | SPNS1 | | |
| GRID2 | STK11 | | |
| HIF1A | TSC1 | | |
| HSPA8 | WDFY3 | | |
